# Supplementary material for: An acid-tolerance response system protecting exponentially growing Escherichia coli
Source: Nat Commun. 2020 Mar 20;11:1496. doi: 10.1038/s41467-020-15350-5 (PMC7083825; doi:10.1038/s41467-020-15350-5)
Supplement: Supplementary file 1 — Supplementary Information [file 41467_2020_15350_MOESM1_ESM.pdf]

Supplementary Information

An Acid-tolerance Response System Protecting Exponentially Growing *Escherichia coli*

Xu et al.

Supplementary Figures

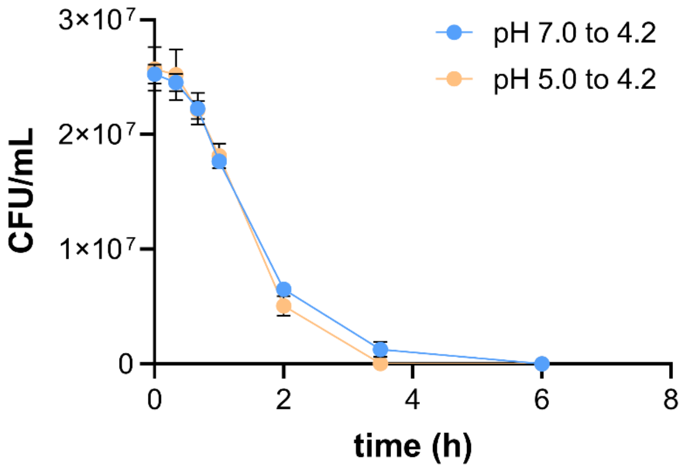

Supplementary Figure 1 Culturability of *E. coli* BW25113 at pH 4.2. BW25113 was grown overnight at pH 7.0 and pH 5.0, and diluted 1:100 into medium E at pH 4.2. Data are presented as mean ± SEM of three independent samples. The source data are provided as a Source Data file.

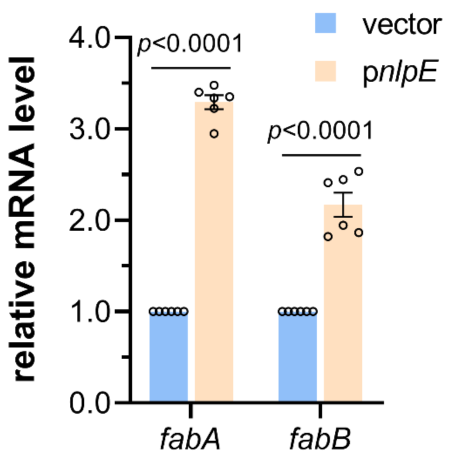

Supplementary Figure 2 Relative mRNA level of *fabA* and *fabB* genes in *fadR fabR* double mutant carrying empty vector or p*nlpE* (n=2 biologically independent samples with 3 technical repeats). Error bars, mean ± SEM. Two-tailed Student's *t*-tests were performed to determine the statistical significance for two group comparisons. The source data are provided as a Source Data file.

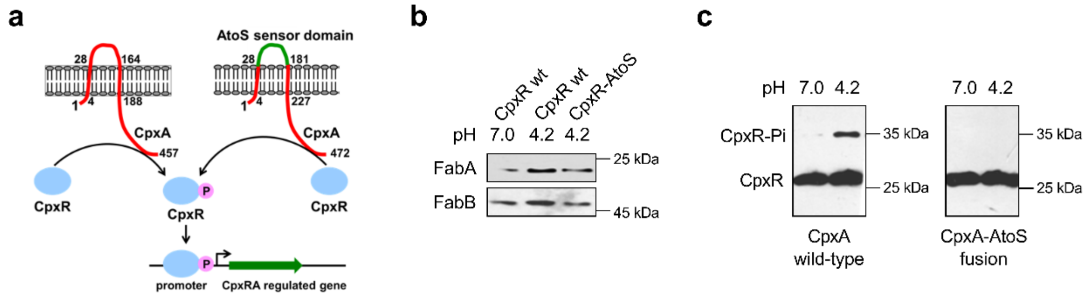

Supplementary Figure 3 CpxA periplasmic domain is required for the sense of acidification. (A) Design and construction of a CpxA-AtoS fusion. The periplasmic domain of CpxA (amino acids 28-164) is replaced by the sensor domain of AtoS (amino acids 38-191, highlighted in green), which sense and response the presence of acetoacetate. (B) Western blot analysis of FabA-His_6 and FabB-His_6 protein in BW25113 strains carrying wild-type CpxA or CpxA-AtoS fusion. (C) *In vitro* analysis of CpxR phosphorylation induced by acidic pH with wild-type CpxA or CpxA-AtoS fusion using reconstituted proteoliposomes. (B) and (C) are representative results from two independent experiments. The source data are provided as a Source Data file.

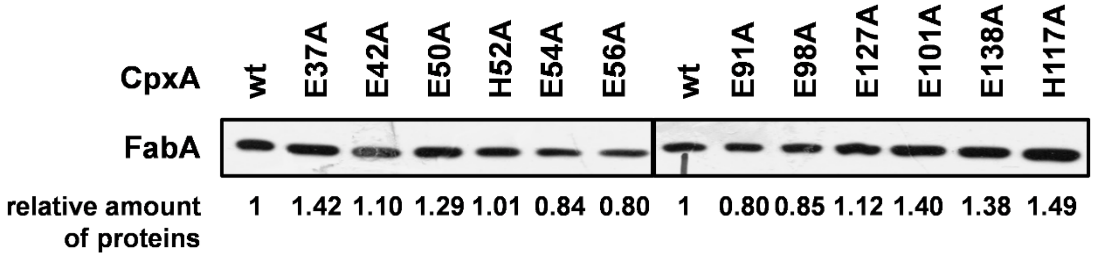

Supplementary Figure 4 Mutations in glutamate and histidine residues in the CpxA periplasmic domain do not impair the sensing of excessive NlpE. FabA-His_6 protein level in BW25113 strains carrying NlpE overexpression plasmid and CpxA variants was determined by Western blot analysis. Results are representative of two independent experiments. The relative amount of protein was determined using ImageJ software. The source data are provided as a Source Data file.

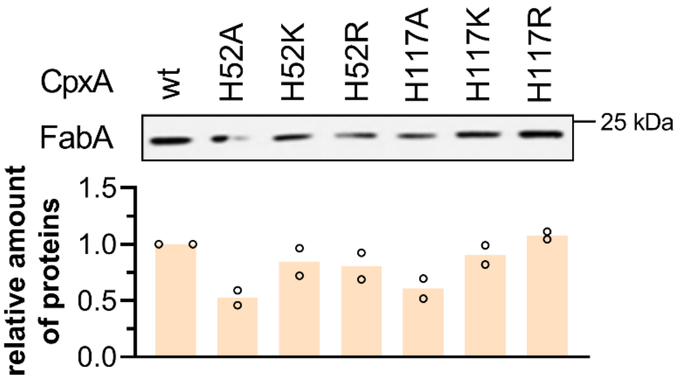

Supplementary Figure 5 Western blot analysis of FabA-His_6 protein in BW25113 strains carrying wild-type CpxA or CpxA mutants in which the histidine at position 52 and 117 was replaced by alanine, lysine, and arginine, respectively, at pH 7.0. The relative amount of protein was determined using ImageJ from two independent experiments. The source data are provided as a Source Data file.

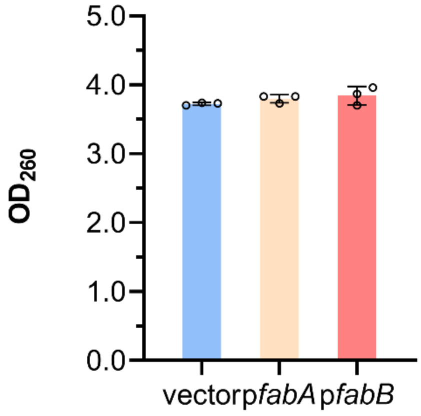

Supplementary Figure 6 The membrane permeability of BW25113 strains carrying empty vector as control, p*fabA*, or p*fabB* after acidic challenge at pH 4.2 for 1 h. It was measured by OD_{260} absorbance. Data are presented as mean ± SEM (n=3 biologically independent samples). The source data are provided as a Source Data file.

```
fabA_BW25113     mvdkresytkedllasgrgelfgakgpqlpapnmlmmdrvvkmtetggnfdkgyveaeld     60
fabA_LT2         mvdkresytkedllasgrgelfgakgpqlpapnmlmmdrvvkmtetggnfdkgyveaeld     60
fabA_2457T       mvdkresytkedllasghgelfgakgpqlpapnmlmidrvvkmtetggnfdkgyveaeld     60
                 *****************:******************:***********************

fabA_BW25113     inpdlwffgchfigdpvmpgclgldamwqlvgfylgwlggegkgralgvgevkftgqvlp     120
fabA_LT2         inpdlwffgchfigdpvmpgclgldamwqlvgfylgwlggegkgralgvgevkftgqvlp     120
fabA_2457T       inpdlwffgchfigdpvmpgclgldamwqlvgfylgwlggegkgralgvgevkftgqvlp     120
                 ************************************************************

fabA_BW25113     takkvtyrihfkrivnrrlimgladgevlvdgrliytasdlkvglfqdtsaf             172
fabA_LT2         tarkvtyrihfkrivnrrlimgladgevlvdgrliytahdlkvglfqdtsaf             172
fabA_2457T       takkvtyrihfkrivnrrlimgladgevlvdcrliytasdlkvglfqdtsaf             172
                 **:**************************** ****** *************
```

Supplementary Figure 7 Sequence alignment of FabA proteins from *E. coli* BW23113, *Salmonella* Typhimurium LT2, and *S. flexneri* 2a str. 2457T. The protein sequences used here are under the Accession Numbers AIN31422, NP_460041, and EFS15200, respectively.
