# Supplementary material for: An acid-tolerance response system protecting exponentially growing Escherichia coli
Source: Nat Commun. 2020 Mar 20;11:1496. doi: 10.1038/s41467-020-15350-5 (PMC7083825; doi:10.1038/s41467-020-15350-5)
Supplement: Supplementary file 3 — Description of Additional Supplementary Files [file 41467_2020_15350_MOESM3_ESM.pdf]

Description of Additional Supplementary Files

File Name: Supplementary Data 1
Description: The strains and plasmids used in this study

File Name: Supplementary Data 2
Description: The primers used in this study
