# Supplementary material for: An acid-tolerance response system protecting exponentially growing Escherichia coli
Source: Nat Commun. 2020 Mar 20;11:1496. doi: 10.1038/s41467-020-15350-5 (PMC7083825; doi:10.1038/s41467-020-15350-5)
Supplement: Supplementary file 6 — Reporting Summary [file 41467_2020_15350_MOESM6_ESM.pdf]

nature research

Corresponding author(s): Guang Zhao, Mo Xian

Last updated by author(s): Feb 27, 2020

Reporting Summary

Nature Research wishes to improve the reproducibility of the work that we publish. This form provides structure for consistency and transparency in reporting. For further information on Nature Research policies, see Authors & Referees and the Editorial Policy Checklist .

Statistics

For all statistical analyses, confirm that the following items are present in the figure legend, table legend, main text, or Methods section.

n/a	Confirmed	
☐	☒	The exact sample size (*n*) for each experimental group/condition, given as a discrete number and unit of measurement
☐	☒	A statement on whether measurements were taken from distinct samples or whether the same sample was measured repeatedly
☐	☒	The statistical test(s) used AND whether they are one- or two-sided<br>*Only common tests should be described solely by name; describe more complex techniques in the Methods section.*
☒	☐	A description of all covariates tested
☒	☐	A description of any assumptions or corrections, such as tests of normality and adjustment for multiple comparisons
☐	☒	A full description of the statistical parameters including central tendency (e.g. means) or other basic estimates (e.g. regression coefficient) AND variation (e.g. standard deviation) or associated estimates of uncertainty (e.g. confidence intervals)
☒	☐	For null hypothesis testing, the test statistic (e.g. *F*, *t*, *r*) with confidence intervals, effect sizes, degrees of freedom and *P* value noted<br>*Give P values as exact values whenever suitable.*
☒	☐	For Bayesian analysis, information on the choice of priors and Markov chain Monte Carlo settings
☒	☐	For hierarchical and complex designs, identification of the appropriate level for tests and full reporting of outcomes
☒	☐	Estimates of effect sizes (e.g. Cohen's *d*, Pearson's *r*), indicating how they were calculated

Our web collection on statistics for biologists contains articles on many of the points above.

Software and code

Policy information about availability of computer code

Data collection	Applied Biosystems QuantStudio 1 for qRT-PCR, and Agilent GC-MS 7890A-5975C with HP-INNOWax Column for fatty acids analysis.
Data analysis	ImageJ software (version 1.52a) for analysis of western blot results. Clustal Omega (https://www.ebi.ac.uk/Tools/msa/clustalo) for sequence alignment.

For manuscripts utilizing custom algorithms or software that are central to the research but not yet described in published literature, software must be made available to editors/reviewers. We strongly encourage code deposition in a community repository (e.g. GitHub). See the Nature Research guidelines for submitting code & software for further information.

Data

Policy information about availability of data

All manuscripts must include a data availability statement. This statement should provide the following information, where applicable:

- Accession codes, unique identifiers, or web links for publicly available datasets
- A list of figures that have associated raw data
- A description of any restrictions on data availability

All data that support the findings of this study are available from the corresponding author upon reasonable request. The source data underlying Figs 1a-b, d-g, 2c-f, 3c-g, 4a, c, e-g, 5a-e, and 6a-b and Supplementary Figs 1, 2, 3b-c, 4, 5, and 6 are provided as a Source Data file. The FabA sequences used in this study are under the Accession Numbers AIN31422, NP_460041, and EFS15200, respectively. The genome sequences used in this study are under the Accession Numbers CP009273, NC_003197, NC_002516, NC_011283, NC_013716, CP001235, NC_004741, CP001589, and NC_009778, respectively.

Field-specific reporting

Please select the one below that is the best fit for your research. If you are not sure, read the appropriate sections before making your selection.

☒ Life sciences ☐ Behavioural & social sciences ☐ Ecological, evolutionary & environmental sciences

For a reference copy of the document with all sections, see nature.com/documents/nr-reporting-summary-flat.pdf

Life sciences study design

All studies must disclose on these points even when the disclosure is negative.

Sample size	The acid tolerance assay, membrane property assay and 3-hydroxypropionate production were performed in triplicate. Western blot and fatty acid composition assay were from two or three independent biological samples. Two independent biological samples with three technical repeats for each sample were performed for each qRT-PCR analysis. For mouse survival passage experiments, three independent trials with six mice in each trial were performed for each strain. Sample size was determined based on the previous experience, and it is sufficient to confirm that results did not vary and were consistent.
Data exclusions	No data were excluded for the analyses.
Replication	Number of replicates for respective analyses were described in the manuscript.
Randomization	Bacterial cultures were randomly assigned to treatment under different pH, and BALB/c mice were randomly divided into several groups for the mouse survival passage experiments.
Blinding	The blinding is not relevant to our study, as our study was performed in E. coli.

Reporting for specific materials, systems and methods

We require information from authors about some types of materials, experimental systems and methods used in many studies. Here, indicate whether each material, system or method listed is relevant to your study. If you are not sure if a list item applies to your research, read the appropriate section before selecting a response.

Materials & experimental systems

n/a	Involved in the study	
☐	☒	Antibodies
☒	☐	Eukaryotic cell lines
☒	☐	Palaeontology
☐	☒	Animals and other organisms
☒	☐	Human research participants
☒	☐	Clinical data

Methods

n/a	Involved in the study	
☒	☐	ChIP-seq
☒	☐	Flow cytometry
☒	☐	MRI-based neuroimaging

Antibodies

Antibodies used	Anti-6X His Tag antibody [GT359] (HRP), mouse monoclonal, Abcam catalog No. ab184607, 1:10000 diluted
Validation	Antibody was validated by manufacturers (WB, https://www.abcam.com/6x-his-tag-antibody-gt359-hrp-ab184607.html) and titrated in our laboratory prior to its use.

Animals and other organisms

Policy information about studies involving animals; ARRIVE guidelines recommended for reporting animal research

Laboratory animals	six-week-old female BALB/c mice were used.
Wild animals	This study did not involve wild animals.
Field-collected samples	This study did not involve samples collected from the field.
Ethics oversight	The animal experiments were performed according to the standards set forth in the Guide for the Care and Use of Laboratory Animals (National Institutes of Health, 1985). Experimental protocols were approved by the Institutional Animal Care Committee at Qingdao Institute of Bioenergy and Bioprocess Technology.

Note that full information on the approval of the study protocol must also be provided in the manuscript.
